# Early handling attenuates enhancement of glucocorticoid receptors in the prefrontal cortex in an animal model of post-traumatic stress disorder

**DOI:** 10.1186/2045-5380-3-22

**Published:** 2013-12-02

**Authors:** Sophie A George, Stephanie A Stout, Melissa Tan, Dayan Knox, Israel Liberzon

**Affiliations:** 1 Department of Psychiatry, University of Michigan, 4250 Plymouth Road, Ann Arbor, MI 48109, USA; 2 Ann Arbor Veterans Affairs Hospital, 2215 Fuller Road, Ann Arbor, MI 49105, USA; 3 Present address: Department of Psychology, University of Delaware, 108 Wolf Hall, Newark, DE 19716, USA

**Keywords:** Hippocampus, HPA axis, Maternal care, Single prolonged stress, Stress

## Abstract

**Background:**

Changes in glucocorticoid receptors (GRs) have been implicated in the pathogenesis of stress related psychiatric disorders such as depression and post-traumatic stress disorder (PTSD). Abnormal adaptation of the stress-response system following traumatic stress can lead to an altered hypothalamic-pituitary-adrenal axis that may contribute to PTSD development. Indeed, elevated GR expression in the hippocampus and prefrontal cortex linked to PTSD-like characteristics have been reported in the validated animal model of PTSD, single-prolonged stress. These findings implicate increased levels of GRs in the development of post-traumatic psychopathology and suggest that exploration of GR-targeted interventions may have potential for PTSD prevention. Early handling during the neonatal phase alters GR expression and is proposed to confer resilience to stress. We therefore examined the effects of combined early handling and single prolonged stress treatments on GR expression.

**Methods:**

Timed pregnant dams gave birth to pups that were subjected to early handling (n = 11) or control (n = 13) procedures during the neonatal phase. At postnatal day 45 animals underwent single prolonged stress or a control procedure. Rats were euthanized one day later and GR levels were assayed using western blot electrophoresis.

**Results:**

Single prolonged stress exposure enhanced GR expression in the hippocampus and prefrontal cortex. Early handling treatment protected against single prolonged stress-induced enhancement of GR expression in the prefrontal cortex, but not in the hippocampus.

**Conclusions:**

These data are a first step in highlighting the importance of targeting GR systems in prevention/resilience and may suggest that preventive strategies targeting GR upregulation might be particularly effective when prefrontal rather than hippocampal GRs are the target.

## Background

Stress initiates a cascade of neuroendocrine events in the hypothalamic-pituitary-adrenal (HPA) axis, which ultimately leads to increased secretion of the glucocorticoid hormone cortisol from the adrenal glands. Activity of the HPA axis is tightly controlled through complex regulatory mechanisms of glucocorticoid negative feedback. Glucocorticoids regulate the secretion of corticotropin-releasing factor and adrenocorticotropic hormone, from the hypothalamus and pituitary, respectively [1-4]. In addition, receptor sites within the hippocampus and prefrontal cortex play an important role in the regulation of HPA axis activity [2,5]. Following chronic or traumatic stress, inappropriate adaptation of the HPA axis can lead to pathological states; specifically, changes in glucocorticoid receptors (GRs) have been implicated in the pathogenesis of stress related psychiatric disorders such as post-traumatic stress disorder (PTSD) [6] and symptoms of PTSD are believed to reflect trauma-induced changes that lead to long-term dysfunctional stress regulation [7-9].

PTSD is characterized by increased cortisol suppression to dexamethasone, believed to result from an increased number or sensitivity of GRs [10]. Recently, in a prospective study, van Zuiden et al. reported higher levels of GR as a risk factor for subsequent development of PTSD in a sample of soldiers [11,12]. Findings from animal models further support changes in GR as the potential mechanism for the development of PTSD symptoms. In addition to reproducing cardinal symptoms of PTSD, such as hyperarousal and elevated fast feedback of the HPA axis [13-16], increased GR levels have been found in the single prolonged stress (SPS) [16-18] and predator exposure models in the hippocampus and prefrontal cortex [19]. In concert, pretreatment with GR antagonists prevents PTSD-like phenotypes in both SPS and predator exposure models [14,20]. Furthermore, in a recent “dismantling” study in which full SPS (involving restraint, forced swim, and ether exposure) was compared to the effect of different components of SPS (i.e., two of three stressors), only those animals that were exposed to the full SPS procedure and demonstrated the greatest degree of upregulation of GR in the hippocampus and prefrontal cortex, exhibited deficits in retention of extinction memories – a mechanism that is proposed to contribute to an inability to retain new safe memories and prevent recovery from trauma [19,21,22]. Together, these findings implicate altered GRs in the development of some aspects of post-traumatic psychopathology, and suggest that exploration of GR-targeted interventions may have potential for PTSD resilience/prevention.

Levine [23-25], and subsequently others (e.g., [26]), demonstrated that glucocorticoid responses to stress were modulated by early life environmental events and could result in stable changes to HPA axis reactivity, most notably via alterations in GR gene expression in the hippocampus and frontal cortex [27]. Early handling (EH), which involves brief daily separation from the mother during the neonatal phase is one such manipulation that has a documented effect on GR expression. EH increases the frequency of maternal behaviors [28,29] and thus increases GR expression and confers resilience to later stress [30,31]. Meany et al. demonstrated that EH enhances the availability of GRs [32], which in turn attenuates stress-induced HPA axis responsivity, as evidenced by attenuated glucocorticoid release in response to stress and reduced anxiety-like behaviors in adulthood [23,27,30,32].

While a number of previous studies have demonstrated that EH can attenuate the effects of chronic stress on inducing HPA axis reactivity [33-35], the effects of EH in animal models of PTSD have not been examined. Given the documented role of GR upregulation in the etiology of PTSD and the demonstration that “traumatic” stress as described in the SPS model increases GR expression, we hypothesized that EH would protect against the GR enhancement that develops following SPS. The goal of this study was to examine the combined effects of EH and single prolonged stress on GR expression. We chose to examine GR changes in the hippocampus and prefrontal cortex because of their documented role in the protective effect of EH [27], as well in the development of SPS-induced changes following traumatic stress [16,19].

## Methods

### Animals

Timed-pregnant dams (Charles River, Portage, MI, USA) were delivered to the Veterans Affairs Veterinary Medical Unit at approximately gestation day 16. Dams were singly housed in a temperature and humidity controlled environment, on a 12 hour light–dark cycle, and had *ad lib* access to standard laboratory chow and water. All experimental procedures were approved by the Veteran Affairs Institutional Animal Care Usage Committee and were in accordance with the National Institute of Health Guide for the Care and Use of Laboratory Animals. The day of birth of the litter was marked as postnatal day (PND) 0. Litter sizes varied naturally between 6 and 12, and on PND 2, animals were culled to ensure that equivalent numbers of males and females were present in each litter. The animals in this experiment were drawn from eight litters, and the number of animals in each litter from which data was sampled ranged from 4–12. Pups were subjected to EH or animal facility reared (AFR) treatments [36]. Briefly, EH litters received 15 minutes of daily maternal separation for 21 days. AFR rats were left undisturbed, except for bi-weekly cage maintenance. On PND 23, pups were weaned and housed in same sex sibling pairs.

### SPS and brain homogenate preparation

On PND 45, 24 male Sprague–Dawley rats were assigned to the SPS (AFR = 7, EH = 5) or control (AFR = 6, EH = 6) groups. SPS rats were exposed to two hours of restraint, followed by 20 minutes of forced swimming in a 55 L container. After 15 minutes recuperation rats were exposed to 70 mL of ether in a desiccator until general anesthesia was induced (typically less than five minutes). Rats were then returned to their home cages for a seven day quiescent period. The SPS procedure refers to the application of the three stressors plus the seven day quiescent period. The quiescent period has been demonstrated to be critical for the development of PTSD-like physiological and behavioral abnormalities following SPS [15,37]. Animals assigned to the control group were left undisturbed in their home cages for the duration of SPS.

Following SPS (i.e., 8 days after the application of acute stressors), rats were euthanized by rapid decapitation, their brains were removed, flash frozen in chilled isopentane and stored in a -80°C freezer for later processing. Brains were then thawed to -20°C in a cryostat and the prefrontal cortex was dissected, approximately 1.00 mm anterior to Bregma [38]. The cerebrum was separated from the brain stem, thawed on ice, and the hippocampus was removed. The prefrontal cortex and hippocampus were sonicated separately in homogenization buffer (50 mM Trizma base, 1 mM ethylenediaminetetraacetic acid, 10% sucrose, 4% sodium dodecyl sulfate, 2X protease inhibitor cocktail (Roche USA), pH 7.0 to 7.4), centrifuged at 105,000 x*g* for 45 minutes, homogenates decanted, and protein content determined using a Pierce BCA kit (Sigma-Aldrich, St. Louis, MO, USA). Approximately 40 μg of protein was diluted into a 1X Lamelli sample buffer and stored in a -80°C freezer until the western blot assay was performed.

### Western blot electrophoresis

Western blot for total GR (cytoplasm and nucleus) was adapted from Spencer et al. [39] and conducted as previously described [19]. Briefly, samples heated at 70°C for 7 minutes were electrophoresed on 7.5% Tris HCl gels (Bio-Rad Laboratories, Inc., Hercules, CA, USA) along with a molecular weight ladder (Li-COR, Lincoln, NE, USA). Proteins in gels were transferred onto nitrocellulose membranes and blocked in blocking buffer (BB) (5% non-fat milk and 0.05% Tween-20 in tris-buffered saline (TBS)). Nitrocellulose membranes were then probed for GR by incubating membranes with a rabbit polyclonal GR antibody (Santa Cruz Biotechnology Inc., Santa Cruz, CA, USA; M-20, diluted 1:500 in BB) for 2 hours. After several washes in 0.05% Tween-20 in TBS, nitrocellulose membranes were incubated with an IRDye 800 conjugated anti-rabbit IgG secondary antibody (Li-COR, diluted 1:2,000 in BB) for 1 hour. Nitrocellulose membranes were then rinsed with TBS and scanned using a Li-COR Odyssey Scanner for visualization of GR bands.

After probing nitrocellulose membranes for GR, the same membranes were probed for actin related protein (Arp) which was used as the reference protein as previously described [40]. Nitrocellulose membranes were incubated with a rabbit polyclonal Arp antibody (Santa Cruz Antibodies, Arp-2, diluted 1:2,000 in BB), washed in 0.05% Tween-20 in TBS, and then incubated with the secondary antibody (Li-COR, 1:8,000 in BB). Nitrocellulose membranes were rinsed with TBS and scanned in a Li-COR Odyssey scanner in order to visualize Arp bands.

Images of scanned nitrocellulose membranes were analyzed using Odyssey software (Li-COR). The integrated intensity of the GR and Arp bands were expressed as a ratio (GR/Arp) and used as a relative measure of GR levels. Each gel contained representative samples from each of the treatment groups (Additional file 1). Samples were initially run in duplicate, but after a small coefficient of variance was established, single samples were run subsequently. GR levels were subjected to two factor analysis with the factors neonatal treatment (EH vs. AFR) and stress treatment (SPS vs. control). GR in the hippocampus and prefrontal cortex were analyzed separately. Main and simple effects were analyzed using analysis of variance (ANOVA), while main and simple comparisons were analyzed using *t*-test with a Bonferroni correction where necessary. Criterion of significance for all tests was set at *P* <0.05.

## Results

**Figure 1:**
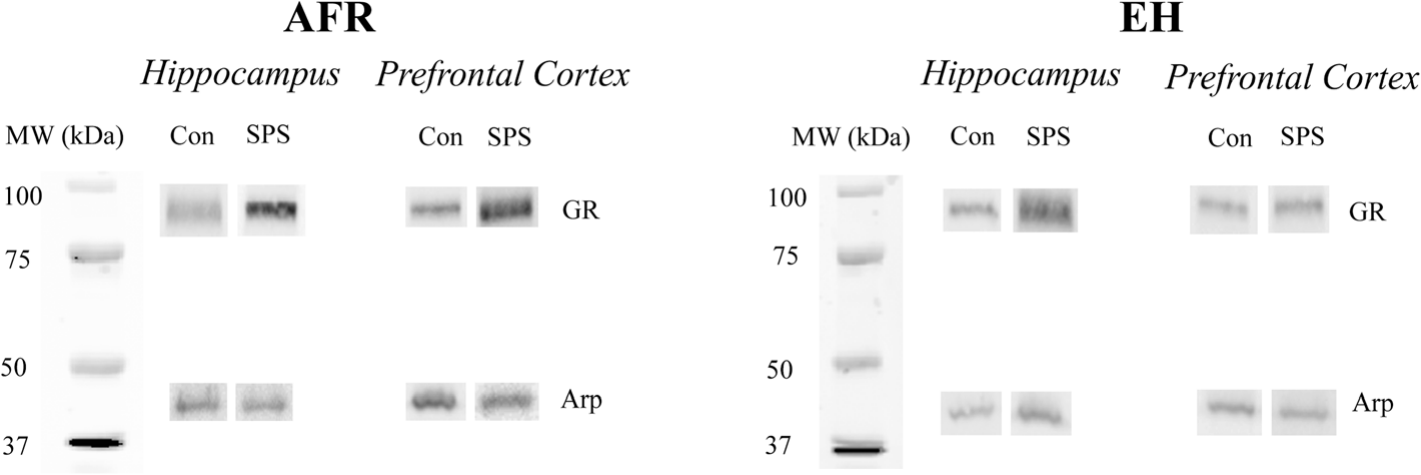
**Representative protein bands from all treatment groups in this study.** MW: Molecular weight markers; AFR: Animal facility reared; EH: Early handling; SPS: Single prolonged stress; Con: Control; GR: Glucocorticoid receptor; Arp: Actin related protein.

Prominent bands were observed between the 100 kDa and 75 kDa molecular weight markers for GR, and 50 kDa and 37 kDa for Arp in both hippocampus and prefrontal cortex (Figure 1). These bands correspond closely to previously determined locations for GR and Arp using the primary antibodies described in the Methods section.

**Figure 2:**
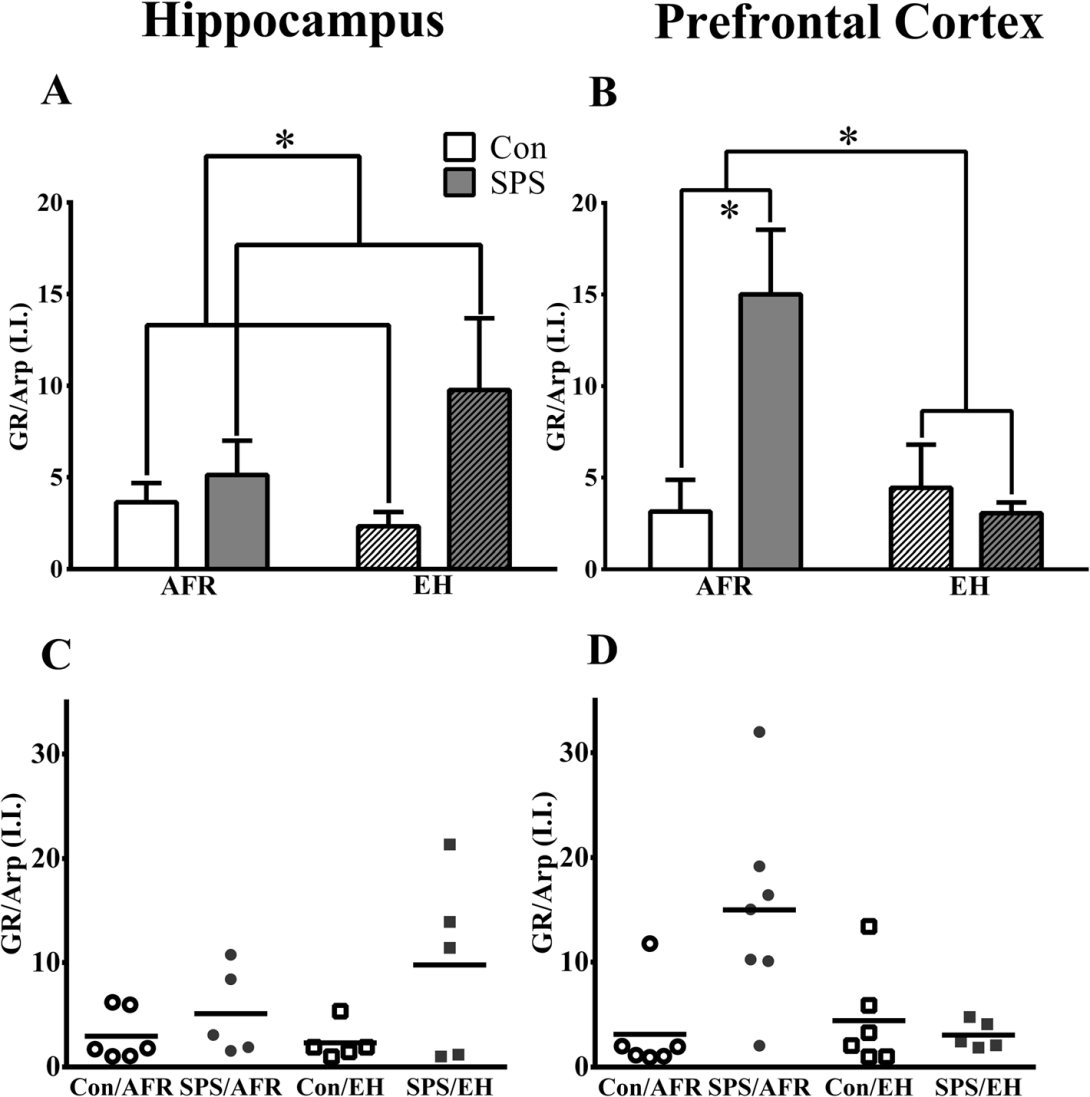
**The effects of early handling (EH) and single prolonged stress on mean relative glucocorticoid levels in the (A) hippocampus and (B) prefrontal cortex. (C,D)** Scatter plots showing individual data points from animals included in this experiment. *P <0.05. AFR: Animal facility reared; EH: Early handling; SPS: Single prolonged stress; Con: Control; GR: Glucocorticoid receptor; Arp: Actin related protein; I.I.: Integrated intensity.

An ANOVA of GR expression in the prefrontal cortex revealed a significant SPS × EH interaction (F_(1,20)_ = 7.077, *P* = 0.015). Post hoc comparisons revealed higher GR signal in SPS animals in comparison to controls in AFR treated groups (t_(11)_ = 2.856, *P* = 0.016), but this effect was not present in the EH exposed groups (t_(9)_ = 0.626, *P* = 0.547), suggesting that SPS-induced enhancement of GR expression in prefrontal cortex was effectively prevented by EH. An ANOVA of hippocampal GR revealed a significant main effect of SPS (F_(1,17)_ = 4.929, *P* = 0.04) with higher GR signal in SPS-exposed animals; however, there was no SPS × EH interaction (F_(1,17)_ = 1.487, *P* = 0.239) or main effect of EH (F_(1,17)_ = 0.851, *P* = 0.369), suggesting that EH did not attenuate SPS-induced increases in GR expression in the hippocampus. These data are illustrated in Figure 2.

## Discussion

In the present experiments SPS enhanced GR expression in the hippocampus and prefrontal cortex, replicating findings previously reported by ourselves and others [16,18,19]. EH, on the other hand, attenuated SPS-induced enhancement of GR in the prefrontal cortex, suggesting that EH may be protective against some of the SPS-induced changes implicated in PTSD pathophysiology. Interestingly, while affecting GR in prefrontal cortex, EH did not attenuate SPS-induced enhancement of GR in the hippocampus suggesting that there are regional differences in GR expression following combined effects of early life environment and stressors experienced in adulthood. It has long been suggested that early life experiences may lead to developmental changes that result in stable alterations to HPA axis and potentially confer resilience to later stress. To our knowledge, this is the first report of the combined effects of early life experiences and later “traumatic” stress on GR expression. Given the established role of GR in HPA axis regulation and stress reactivity [30,31] as well as in the pathophysiology of the SPS model of PTSD [16], these findings may have some implications for understanding mechanisms of resilience to traumatic stress, and of the regional differences that may be critical in moderating the protective effect of early life experiences to later life stressors. While intriguing, the functional implications of these GR expression changes will need to be examined in order to further establish the significance of this finding. In addition, given the heterogeneity of the prefrontal cortex, in particular with respect to regulation of stress reactivity conferred by environmental influences [41], it would be interesting to further examine whether different subregions of the prefrontal cortex contribute differentially to the effect reported herein.

Differential changes in GR expression between frontal cortex and hippocampus following stress manipulations have been previously reported by our laboratory [19] as well as by others. Indeed, in Meaney’s seminal paper in which the effect of EH on GR in the frontal cortex and hippocampus is first described, GR in the hippocampus was increased in EH animals compared to non-handled controls irrespective of post-weaning housing conditions [32]. However, this was not the case in the frontal cortex in which post-weaning housing condition moderated GR expression. These data suggest that hippocampal changes in GR may be more stable and enduring than those in the frontal cortex, resonating with our own finding, in which hippocampal GRs were found to be less sensitive to environmental effects than GRs in the prefrontal cortex.

The precise functional role of hippocampal and prefrontal cortex GRs are not known, although a wealth of data suggests that receptor sites within the hippocampus and prefrontal cortex play an important role in the regulation of HPA axis activity [2,5]. Recent data from our own laboratory, in which full SPS (comprised of all three stressors) was compared to partial SPS procedures (e.g., restraint + ether or forced swim + restraint), demonstrated that exposure to ether alone was sufficient to alter prefrontal GR levels, while multiple combined stressors were required to alter GR levels in the hippocampus. Moreover, the behavioral data from this study indicated that the combined effect of serial exposure to all three stressors (restraint, forced swim and ether) was required in order to observe extinction retention deficits. These results suggest that the mere enhancements in GR expression in the hippocampus and prefrontal cortex might be insufficient to lead to PTSD-relevant behavioral deficits, but “threshold” change in these regions is required for SPS-induced extinction retention deficits to manifest. Together with the present data, these findings suggest that the ability of EH to attenuate SPS-induced enhancement in prefrontal GR levels should be interpreted with caution as they may not necessarily translate to resilience in PTSD-relevant behavioral outcomes. Addressing this question directly, for instance by examining the effect of EH on extinction retention deficits in SPS animals, will be an important goal of future studies.

Interestingly, in these experiments we did not detect effects of EH alone on total GR expression. This is in contrast to the findings reported by Meaney et al., in which EH was found to increase baseline levels of unbound cytoplasmic GR. There are several possibilities that may explain this apparent discrepancy; EH effects are known to be mediated by FKBP5 protein modulating GR sensitivity to ligands [42]. Thus, when FKBP5 is bound to GR, binding of glucocorticoids to GRs is reduced. It is therefore possible that EH could increase GR sensitivity by attenuating FKBP5-GR binding. Because radioimmunoassays are typically used to assay unbound cytoplasmic GR, these assays rely on protein-ligand binding and therefore a treatment that increases GR sensitivity could be interpreted as an increase in unbound cytoplasmic GR. Thus, the differing approaches to measuring GR levels may explain these apparently contradictory findings. Alternatively, there were a number of other methodological differences that may underlie the difference in baseline EH findings between the two studies. For example, different strains of rat were used and the age at which GR was measured was different, as were post-weaning housing conditions, all of which have been suggested to impact GR expression [32].

Interestingly, in Meaney’s model [32], increases in GR expression are interpreted as functionally beneficial, with EH increasing GR expression and conferring later resilience to stress. Accordingly, prolonged maternal separation, which reduces GR expression, is proposed to have adverse consequences, resulting in vulnerability to later stress. Conversely, our data suggest that GR increases following SPS relate to greater functional impairment [19]. The differences in the developmental stages at which GR changes are initiated may be critical to the behavioral impact of GR changes, explaining the seemingly conflicting results. The present data shows that EH prevents trauma-induced increases in GR in adult fully-grown animals, thus suggesting that early life EH protects against later increases in GR, possibly because of a more efficient negative feedback system which clamps down the HPA axis response following traumatic stress. Critically, both studies confirm EH results in changes in GR expression that likely result in resilience but further research is clearly needed to examine the precise mechanisms by which EH modulates GR expression following different stressors and in different brain regions.

## Conclusions

While a number of previous studies have demonstrated that EH can attenuate the effects of chronic stress on inducing HPA axis reactivity, to our knowledge, this is the first study to examine the effects of EH in an animal model of PTSD. The data reported here suggest that the early life environment may have an important role in later responses to traumatic stress, and suggest that regional differentiation in GR expression may be important characteristic of the effects. These data, while limited to a measure of protein expression, underscore the importance of targeting GR systems in prevention/resilience and suggest that preventive strategies targeting GR upregulation may be more effective when prefrontal rather than hippocampal GRs are the target.

## Abbreviations

AFR: Animal facility reared; Arp: Actin related protein; BB: Blocking buffer; EH: Early handling; GR: Glucocorticoid receptor; HPA: Hypothalamic-pituitary-adrenal axis; PND: Postnatal day; PTSD: Post-traumatic stress disorder; SPS: Single prolonged stress; TBS: Tris-buffered saline.

## Competing interests

The authors declare that they have no competing interests.

## Author contributions

SG was the primary writer of the manuscript. She also contributed to data collection, statistical analyses and interpretation of the data. DK contributed to the conception and design of the experiment, statistical analyses and interpretation of the data. He supervised the data acquisition and substantially contributed to the drafting and revision of the manuscript. SS and MT were primarily responsible for acquisition of data. They conducted all early handling protocols and stress procedures and carried out initial statistical analyses. IL bore overall responsibility for conception and design of the study, and interpretation of the data. He made critical revisions to the manuscript. All authors read and approved the final manuscript.

## Supplementary Material

Additional file 1 **Representative example of a western blot assay.** The data presented in this manuscript were obtained from a larger research project that examined the effects of a number of neonatal treatments and single prolonged stress on GR expression in the hippocampus and prefrontal cortex. This additional data file shows a representative example of a western blot assay. Click here for file
