# Supplementary material for: Early handling attenuates enhancement of glucocorticoid receptors in the prefrontal cortex in an animal model of post-traumatic stress disorder
Source: Biol Mood Anxiety Disord. 2013 Dec 2;3:22. doi: 10.1186/2045-5380-3-22 (PMC4175489; doi:10.1186/2045-5380-3-22)
Supplement: Additional file 1 — Representative example of a western blot assay. The data presented in this manuscript were obtained from a larger research project that examined the effects of a number of neonatal treatments and single prolonged stress on GR expression in the hippocampus and prefrontal cortex. This additional data file shows a representative example of a western blot assay. [file 2045-5380-3-22-S1.docx]

Representative example of a western blot assay

Using procedures described in the Methods, nitrocellulose membranes were first probed for glucocorticoid receptor (GR) protein, and scanned using the Li-COR Odyssey scanner to visualize molecular weight (MW) markers and obtain the integrated intensity of the GR bands. Probing the nitrocellulose membrane for GR resulted in a single band in each lane that occurred between the 100 kDa and 75 kDa molecular weight markers. The location of this band corresponds closely with the molecular weight of GR (α isoform = 95 kDa, β isoform = 90 kDa) (Panel A). After probing for GR, the same nitrocellulose membrane was then probed for mineralocorticoid receptor (MR) protein. This resulted in three bands (Panel B). The first and third bands are non-specific, and the second band is the putative MR band (anti-MR antibody, Santa Cruz Biotechnology Inc.). The specific MR band was not reliably observed in Western Blot assays, therefore we did not analyze the integrated intensity of this band. Finally, nitrocellulose membranes were probed for actin related protein (Arp). Arp was used as a reference protein. Probing nitrocellulose membranes for Arp resulted in the addition of another band, which occurred between the 50 kDa and 32 kDa MW markers (Panel C). This corresponds closely to the molecular weight of Arp (43 kDa). Samples obtained from the hippocampus and prefrontal cortex of animals were assayed. The data presented in this manuscript were obtained from a larger research project that examined the effects of a number of neonatal treatments and single prolonged stress on GR expression in the hippocampus and prefrontal cortex. Samples were initially run in duplicates to reduce measurement errors, however after it was determined that the coefficient of variance was very small, samples were run singly to allow representative samples from each treatment group and from two different brain regions on every gel. Data from aninals in treatment groups not decribed in this manuscript are represented by a dash.


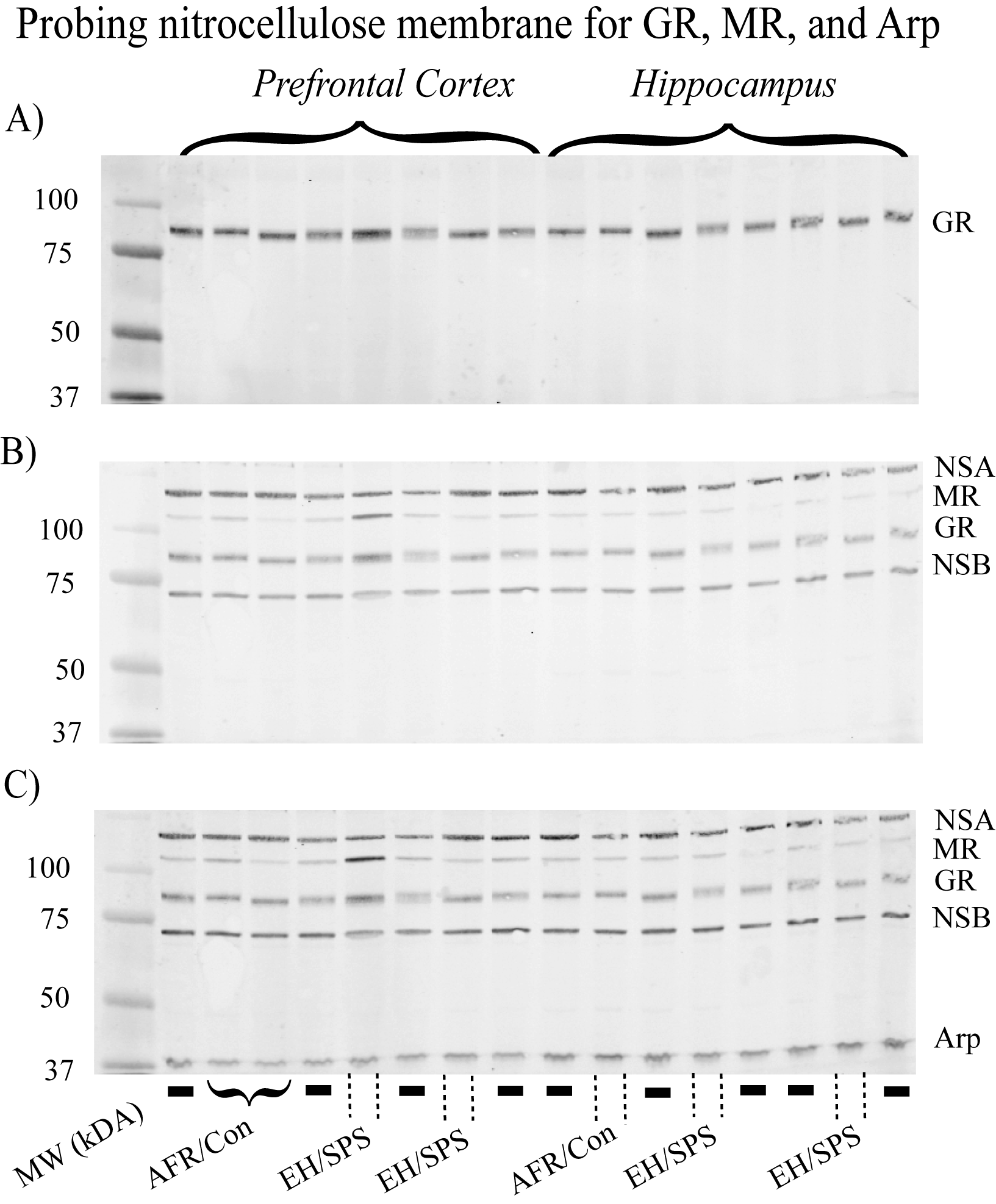


AFR – animal facility reared, EH – early handling, GR – glucocorticoid receptor, MR – mineralocorticoid receptor, MW – molecular weight, NSA, NSB – non-specific A and B.
